# Supplementary material for: Assessment of Periprostatic and Subcutaneous Adipose Tissue Lipolysis and Adipocyte Size from Men with Localized Prostate Cancer
Source: Cancers (Basel). 2020 May 28;12(6):1385. doi: 10.3390/cancers12061385 (PMC7352157; doi:10.3390/cancers12061385)
Supplement: Supplementary file 1 [file cancers-12-01385-s001.pdf]

Supplemental Materials

Assessment of Periprostatic and Subcutaneous Adipose Tissue Lipolysis and Adipocyte Size from Men with Localized Prostate Cancer

Dushan Miladinovic, Thomas Cusick, Kate L. Mahon, Anne-Maree Haynes, Colin H. Cortie, Barbara J. Meyer, Phillip D. Stricker, Gary A. Wittert, Lisa M. Butler, Lisa G. Horvath and Andrew J. Hoy

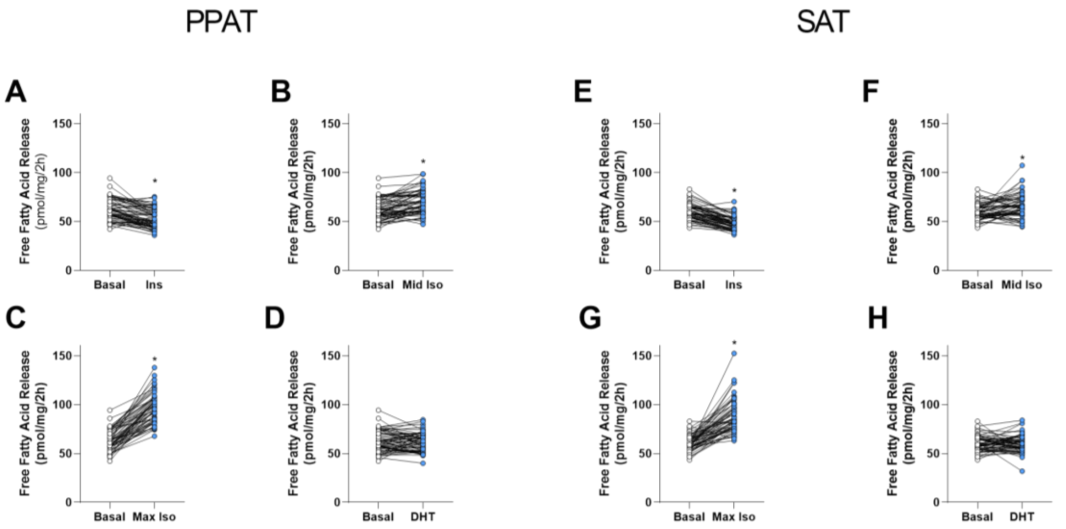

Figure S1. Periprostatic and subcutaneous adipose tissue hormone stimulate lipolysis. (**A**) Insulin (10 nM; Ins), (**B**) mid- (10 nM; mid Iso) and (**C**) max-isoproterenol (1 μM; max Iso), (**D**) dihydrotestosterone (1 nM; DHT) stimulated free fatty acid release from periprostatic adipose tissue (PPAT). (**E**) Insulin (10 nM; Ins), (**F**) mid- (10 nM; mid Iso) and (**G**) max-isoproterenol (1 μM; max Iso), (**H**) dihydrotestosterone (1 nM; DHT) stimulated free fatty acid release from subcutaneous adipose tissue (SAT). All data was expressed as mean ± SEM. * $p < 0.05$ by a non-parametric Mann Whitney *U* test. *N* = 60.

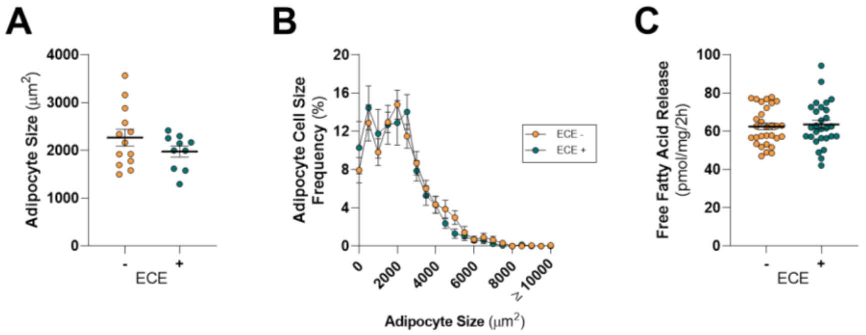

Figure S2. Comparison of periprostatic adipose tissue adipocyte size and lipolysis in men with extracapsular extension (ECE) or not. (**A**) Mean and (**B**) distribution of adipocyte size of periprostatic adipose tissue (PPAT), and (**C**) basal fatty acid release rates and dichotomized into minus or plus extracapsular extension (ECE). All data was expressed as mean ± SEM. (A & B) Extracapsular Extension (−) *n* = 13, Extracapsular Extension (+) *n* = 9. (C) Extracapsular Extension (−) *n* = 30, Extracapsular Extension (+) *n* = 28

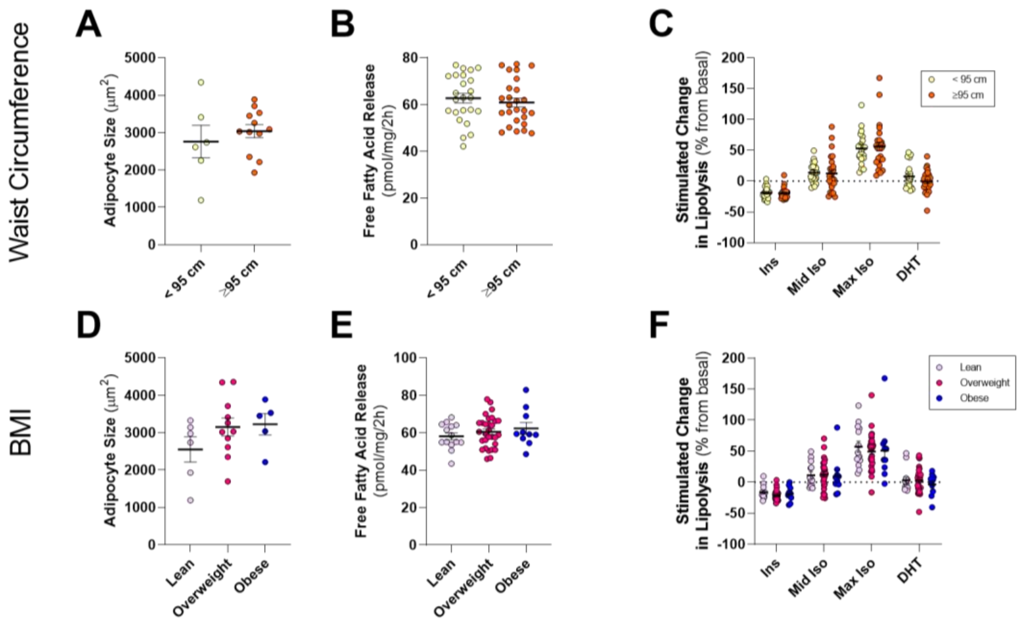

Figure S3. Comparison of subcutaneous adipose tissue adipocyte size and lipolysis in lean or obese men with prostate cancer. (**A**) Mean adipocyte size of subcutaneous adipose tissue (SAT) dichotomized into waist circumference < 95 cm (normal) and ≥ 95 cm (high) groups; (**B**) Basal fatty acid release rates and (**C**) change in basal free fatty acid release from SAT in response to 10 nM insulin (Ins), 10 nM (mid Iso) and 1 µM (max Iso) isoproterenol, and 1 nM dihydrotestosterone (DHT) dichotomized into waist circumference < 95 cm (normal) and ≥ 95 cm (high) groups. (**D**) Mean adipocyte size of SAT stratified by BMI into Lean (<25 kg/m²), Overweight (≥25 kg/m², <30 kg/m²) and Obese (≥30 kg/m²) groups; (**E**) Basal fatty acid release rates and (**F**) change in basal free fatty acid release from PPAT in response to 10 nM insulin (Ins), 10 nM (mid Iso) and 1 µM (max Iso) isoproterenol, and 1 nM dihydrotestosterone (DHT) stratified by BMI into Lean, Overweight and Obese groups. All data was expressed as mean ± SEM. (A) <95 cm *n* = 6, ≥95 cm *n* = 12. (B & C) <95 cm *n* = 24, ≥95 cm *n* = 26. (D) Lean *n* = 6, Overweight *n* = 11, Obese *n* = 5. (E & F) Lean *n* = 14, Overweight *n* = 30, Obese *n* = 10.

Table S1. Comparison of the basal secreted fatty acid profile from periprostatic and subcutaneous adipose tissue and published studies reporting intracellular periprostatic fatty acyl species.

	**Current Study**		**Careaga et al 2015**	**Mamalakis et al 2002**
**Fatty Acid (mol %)**	**SAT Secreted**	**PPAT Secreted**	**PPAT Intracellular**	**Prostatic malignancy**
SFA				
14:0	2.26 ± 0.11	2.47 ± 0.08	2.64 ± 0.68	1.35 ± 0.42
16:0	21.54 ± 0.18	22.32 ± 0.18	23.2 ± 1.65	24.4 ± 2.79
18:0	16.50 ± 0.46	15.89 ± 0.31	6.05 ± 0.80	13.2 ± 6.32
MUFA				
16:1 n-7	2.63 ± 0.14	2.46 ± 0.10	4.35 ± 1.24	0.50 ± 0.24
18:1 n-9	30.65 ± 0.61	31.16 ± 0.46	43.3 ± 1.28	34.2 ± 12.0
PUFA *n-6*				
18:2 n-6	12.20 ± 0.21	12.00 ± 0.23	17.4 ± 0.55	8.46 ± 1.88
18:3 n-6	0.32 ± 0.07 *	0.11 ± 0.04	N/R	N/R
20:3 n-6	1.54 ± 0.08 *	1.28 ± 0.05	0.27 ± 0.08	0.95 ± 0.54
20:4 n-6	2.21 ± 0.09 *	1.84 ± 0.06	0.28 ± 0.08	5.99 ± 3.65
22:5 n-6	0.13 ± 0.06	0.04 ± 0.04	N/R	0.12 ± 0.07
PUFA n-*3*				
18:3 n-3	2.31 ± 0.07	2.24 ± 0.06	N/R	0.18 ± 0.08
20:5 n-3	1.10 ± 0.05	1.02 ± 0.04	N/R	0.08 ± 0.06
22:5 n-3	1.83 ± 0.05 *	1.64 ± 0.05	0.18 ± 0.07	0.37 ± 0.17
22:6 n-3	0.58 ± 0.10 *	0.27 ± 0.04	0.14 ± 0.09	1.23 ± 0.64
Summary statistics				
∑ SFA	42.07 ± 0.50	42.71 ± 0.42	32.9 ± 1.86	41.2 ± 9.43
∑ MUFA	35.16 ± 0.75	36.46 ± 0.52	48.7 ± 1.31	39.1 ± 12.8
∑ PUFA	22.77 ± 0.5 *	20.83± 0.37	18.1 ± 2.72	19.7 ± 4.68
∑ n-6	16.95 ± 0.34 *	15.66 ± 0.27	N/R	15.7 ± 3.17
∑ n-3	5.81 ±0.18 *	5.17 ± 0.15	N/R	1.84 ± 0.79
n-3 / n-6 ratio	0.34 ± 0.01	0.33 ± 0.01	N/R	0.11 ± 0.03

Mean ± SEM are shown for SAT and PPAT Secreted, and Mean ± SD for Careaga et al. 2015 and Mamalakis et al. 2020. * $p < 0.05$ vs. PPAT secreted by a Student *t*-test. SFA: saturated fatty acid; MUFA: monounsaturated fatty acid; N/R: Not Reported; PUFA: polyunsaturated fatty acids.

© 2020 by the authors. Submitted for possible open access publication under the terms and conditions of the Creative Commons Attribution (CC BY) license (http://creativecommons.org/licenses/by/4.0/).
